# Supplementary material for: Chromothripsis during telomere crisis is independent of NHEJ, and consistent with a replicative origin
Source: Genome Res. 2019 May;29(5):737–49. doi: 10.1101/gr.240705.118 (PMC6499312; doi:10.1101/gr.240705.118)
Supplement: Supplemental Material [file supp_gr.240705.118_Supplemental_file_1.zip › contigs/annotated_contigs/DB55/contig.3.DB55_length_738_mean_cov_8.05420054201.docx]

**DB55_length_738_mean_cov_8.05420054201**

```
CTAGCCAGTTTTCCCAGCACCATTTATTAAATAGGGAATCCTTTCCCCATTGCCTGTTTTTGTCAGGTTTGTCAAAGATCAGATAGTTG
 >chr10:1348745-1349069 - E=4e-180 p=3e-02
TAGATATGCGGCGTTATTTCTGACGGCTCTGTTCTGTTCCATTGATCTATATCTCTGTTTTGGTACCAGTACCATGCTGTTTTGGTACC

AGTACCATGCTGTTTTGGTTACTGTAGCTTTGTAGTATAGTTTGAAGTCAGGTAGTGTGATGCCTCCAGCTTTGTTCTTTTGGCTTAGG

ATTGACTTAGCGATGCGGGCTCTTTTTTGGTTCCATATGAACTTTAAAGTAGTTTT|T|ATCTACTTTCACAAAGTCTGGTCCATTTTT
                                                         >chr12:1850956-1851046 - E=2e-
TAAAATGATTTACCTAAGAAAAAAGAACTCTAGCACTAAAGAAAATCACTTAATTGCA|T|CCAATTCTGTGAAGAAAGTCATTGGTAG
42                                                         >chr9:97233042-97233368 + E=
CTTGATGGGGATGGCATTGAATCTTACCTTGGGCAGTATGGCCATTTTCACGATATTGATTCTTCCTACCCATGAGTATGGAATGTTTT
2e-184
TCCATTTGTTTGTATCCTCTTTTATTTCATGGAGCAGTGGTTTGTAGTTCTCCTTGAAGAGGTCCGATCATTTCTAGCTTTTGATTTAA

AGTGAGAGACATGCAACTCTTCCCTTTACTCAAACCCTTAGAAGCCATTGCAGGGTAATTAATTGGCCTGATATCAATATTGTCATATC

TGAAAGAATGGAGAGGCCCAAAGGAAGGGA
```
